# Imaging a target of Ca^2+^ signalling: Dense core granule exocytosis viewed by total internal reflection fluorescence microscopy

**DOI:** 10.1016/j.ymeth.2008.09.016

**Published:** 2008-11

**Authors:** Magalie A. Ravier, Takashi Tsuboi, Guy A. Rutter

**Affiliations:** a Unit of Endocrinology and Metabolism, University of Louvain Faculty of Medicine, UCL 55.30 Avenue Hippocrate 55, B-1200 Brussels, Belgium; b Department of Life Sciences, Graduate School of Arts and Sciences, The University of Tokyo, 3-8-1, Komaba, Meguro, Tokyo 153-8902, Japan; c Department of Cell Biology, Division of Medicine, Faculty of Medicine, Sir Alexander Fleming Building, Imperial College London, Exhibition Road, London SW7 2AZ, UK

**Keywords:** Exocytosis, Fluorescent protein, Hormone secretion, Live cell imaging, Total internal reflection fluorescence microscopy

## Abstract

Ca^2+^ ions are the most ubiquitous second messenger found in all cells, and play a significant role in controlling regulated secretion from neurons, endocrine, neuroendocrine and exocrine cells. Here, we describe microscopic techniques to image regulated secretion, a target of Ca^2+^ signalling. The first of these, total internal reflection fluorescence (TIRF), is well suited for optical sectioning at cell–substrate regions with an unusually thin region of fluorescence excitation (<150 nm). It is thus particularly useful for studies of regulated hormone secretion. A brief summary of this approach is provided, as well as a description of the physical basis for the technique and the tools to implement TIRF using a standard fluorescence microscope. We also detail the different fluorescent probes which can be used to detect secretion and how to analyze the data obtained. A comparison between TIRF and other imaging modalities including confocal and multiphoton microscopy is also included.

## 1 Introduction

The vast majority of cells in most organisms need to ensure a rapid and continuous delivery of plasma membrane proteins to the cell surface, and the release of soluble proteins and other biological substances, using a process known as constitutive exocytosis. In contrast, a regulated biosynthetic-secretory pathway is only present in cells that are specialized for secretion such as neurons, exocrine, endocrine and neuroendocrine cells. Thus, these cells have sophisticated release mechanism directly tuned by extracellular signals. This process, referred to as regulated exocytosis, ensures the timely delivery of molecules such as peptide hormones and digestive enzymes to fulfill the minute-to-minute requirements of the organism.

Ca^2+^ is the most ubiquitous second messenger found in all cells, and plays a significant role in controlling regulated secretion [1–3]. In some cells including squid giant synapse or pancreatic β-cells, it has been reported that Ca^2+^ channels and vesicles are tightly coupled in space (∼10 nm), whereas in other neurons Ca^2+^ channels and vesicles at the active zone are coupled less closely (∼100 nm), though Ca^2+^ microdomains are still able to trigger exocytosis [4–6]. In this section, we will not describe the techniques to measure Ca^2+^ changes either in the cytosol or in organelles, as other sections of this volume already focus on this point. Instead, we will describe a method to image secretion, a target of Ca^2+^ signalling. Several methods have been developed to measure secretion from cells. As an example, insulin secretion from pancreatic β-cells, containing 1000–5000 cells, has been measured by static incubation or dynamic perifusion [7–9]. However, the latter techniques which are based on measuring secretion with a radioimmunoassay, are not sensitive enough to measure secretion from a cell cluster or from single β-cells. Other approaches have been developed, including amperometry of 5-hydroxytryptamine (5-HT) that is “preloaded” into the vesicles [10,11], Zn^2+^ detection (as Zn^2+^ is co-released with insulin) [12] and capacitance measurements [5]. Unfortunately, none of the techniques above allow the visualisation of exocytosis. This means that important cellular events occurring at the cell surface during exocytosis, such as vesicle movements, recruitment and fusion with the plasma membrane to release the cargo go unobserved.

Optical live cell imaging techniques represent a powerful tool for detecting vesicle movement and trafficking in live cells and have provided significant advances in understanding the molecular mechanisms of regulated hormone secretion in many cell types. In particular, total internal reflection fluorescence (TIRF) microscopy is a powerful optical technique that allows extremely thin optical sectioning (100–200 nm) with excellent signal-to-noise ratios. This technique has been used to measure changes in Ca^2+^ concentration beneath the plasma membrane [13,14], to track protein translocation [15] or to investigate granule behavior immediately adjacent to the plasma membrane [9,14,16,17]. Indeed, it has been used to measure simultaneously, changes in [Ca^2+^]_c_ (wide field) and insulin exocytosis (TIRF) from single human β-cells [18] or microdomains of Ca^2+^ and transmitter release in synaptosomes [14]. Here, we describe the principle, setting up and probes for total internal fluorescence microscopy using chromaffin and islet β-cells as models.

## 2 Description of method

### 2.1 Principle of total internal reflection fluorescence

**Fig. 1:**
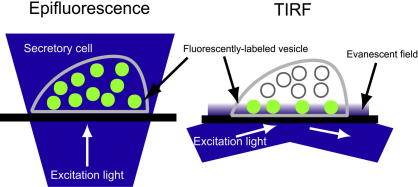
Comparison of epifluorescence versus total internal reflection fluorescence microscopy. The thin layer of illumination is an evanescent field produced by an excitation light beam in a glass cover slip that is incident at a high angle upon the solid-solution interface at which the cells adhere. Thus, an evanescent wave arises on the cell–substrate interface and penetrates a small distance (∼150 nm) into the cells.

The principle of total internal reflection fluorescence (TIRF) microscopy, illustrated in Fig. 1, is based on the excitation of fluorophores by an evanescent field of totally internally reflected light. To achieve total internal reflection, the laser beam is directed obliquely at the interface between two media from a high (coverslip) to a low (cell adherent to the coverslip) refractive index with an incident angle greater than the critical angle of total internal reflection. Total internal reflection sets up a thin layer of light in the cell, called the evanescent field. The strength of this field (termed the evanescent wave) decreases exponentially and its effects extend only a few hundred nanometer into the cell. The portion of the specimen within the evanescent field can therefore be excited to emit fluorescence selectively.

As an example, in Fig. 1, the selective excitation of fluorescent labeled vesicles in a single cell (refractive index, *n*_2_ = 1.38) adherent to a glass coverslip (refractive index, *n*_1_ = 1.53), wave fronts from a laser excitation source pass trough the coverslip and are reflected from the coverslip–cell interface at a critical angle *α*, generating an evanescent wave. Fluorescent labeled vesicles (∼200 nm diameter) at the coverslip interface are excited by the evanescent wave and emit fluorescence which can be recorded, while those located farther away are not excited (Fig. 1, TIRF and Fig. 3B and D). With a conventional fluorescence microscope (Fig. 1, Epifluorescence and Fig. 3A and C) essentially all fluorescent vesicles in the cell are excited, so the emitted fluorescence from the vesicles at the interface of the coverslip is overwhelmed by the considerable number of the other vesicles which are not bound to the glass coverslip.

The critical angle (*α*) at which total internal reflection of a beam of light hitting the interface between two different refractive indexes substances (i.e. coverslip and culture medium for TIRF imaging) is established by:

$$\alpha \geq {\text{sin}}^{-1}\left(\frac{n_{2}}{n_{1}}\right)\text{,}\;n_{1}>n_{2}$$

where, *n*_1_ is the refractive index of the coverslip, *n*_2_ is the refractive index of cells. In our lab, we use an objective lens with a numerical aperture (NA) of 1.45 (PlanAPO 100X NA = 1.45 TIRFM, Olympus) to monitor single vesicle exocytosis [9], thus the critical angle for total internal reflection is:

$$\alpha =\sin^{-1}\left(\frac{1.38}{1.53}\right)=64.41^{\circ }$$

In contrast, the maximal achievable angle for total internal reflection by using this high NA = 1.45 objective lens (i.e. *n*_2_ equals NA of objective lens) with coverslip is:

$$\alpha =\sin^{-1}\left(\frac{1.45}{1.53}\right)=71.39^{\circ }$$

Therefore, the laser beam has to be introduced into the middle of these angles.

Furthermore, the decay length of evanescent field (*d*) can be calculated theoretically using the formula:

$$d=\frac{\lambda }{4\pi \sqrt{n_{2}^{2}\sin^{2}\theta -n_{1}^{2}}}$$

where *θ* is the angle of incidence, *λ* is the wavelength of the laser. For *θ* = 67.5° and *λ* = 488 nm, decay constant (*d*) becomes 81.1 nm, indicating that a layer of 100–200 nm area from the coverslip and cell interface is the illumination area of the evanescent light. Therefore, TIRF microscopes are extremely sensitive to movements of fluorescent objects vertical to the coverslip, as structures brighten when they approach the upper side of the coverslip and dim when they go farther away, resulting in images with very low background fluorescence, nearly no out-of-focus fluorescence, and minimal exposure of cells to light at any other planes in the sample. Further details on TIRF microscopy can be found in several excellent reviews [19–22].

### 2.2 Step-by-step protocol for setting up objective lens type TIRF in standard fluorescence microscope

There are several types of TIRF arrangements, but the most common are those with illumination via a side prism or through a high numerical aperture (NA) objective. The main disadvantage of using the side prism configuration is that the coverslip has to be localized between the objective and the prism. Here, we will focus on the configuration with a high numerical aperture objective, a system we use in routine in our lab. For this configuration, cells are adherent to a coverslip.

Configuring a microscope for objective lens type TIRF excited by a laser beam can be done either by using fairly simple custom built (see below and [16,22–27]) or commercially available, accessories (for example, Olympus IX2-RFAEVA-2).

#### 2.2.1 Equipment

The system is composed of an inverted fluorescence microscope equipped with an epi-illumination dichroic mirror and barrier filter that is appropriate for the selected laser. One significant problem when acquiring time lapse data in TIRF is focus drift. As the region of fluorescence excitation is thin (<150 nm), this can introduce significant artifacts. Companies are starting to sell components to avoid this problem. As an example, Nikon have developed a Perfect Focus System which automatically detects the surface of the coverslip optically and corrects focus to compensate.

When setting up a TIRF system, critical points are in the selection of: (a) the high numerical aperture objective, (b) laser source and (c) camera.

##### 2.2.1.1 Objective with high numerical aperture

**Fig. 2:**
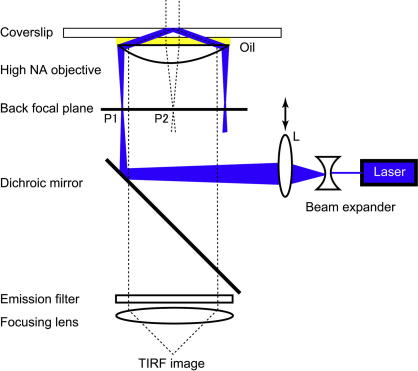
Arrangement for objective lens-type TIRF in an inverted microscope. Laser illumination through a side port requires a dichroic mirror cube facing the side. At the P1 position, the laser beam is focused to lead the critical angle propagation into the coverslip (total internal reflection). Moving the lens L transversely changes the angle of incidence, thus the laser beam moves from P1 (TIRF) position to P2 position (epifluorescence). This system allows to switch between both mode of illumination (TIRF and epifluorescence).

To build an objective lens type TIRF microscopy system, the incident laser light has to pass through the edge of the high numerical aperture (⩾1.45) objective lens. This can be achieved simply by placing the focused incident laser light at edge of back focal plane of the high NA objective lens [25] (Fig. 2).

The highest aperture available is an Olympus 100× NA = 1.65 [28,29]. However, the NA = 1.65 objective requires the use of expensive 1.78 refractive index coverslips and special *n* = 1.78 oil which is volatile and leaves a crystalline residue. Moreover, a special coating of the glass coverslip is required which could be toxic for some cell types (i.e. for primary cells, personal observation).

Fortunately, objectives are now available with NA = 1.45, to avoid the above problems. We will not enumerate all of the existing objectives because they are available from several companies (Olympus, Zeiss, Leica, and Nikon). Basically, these objectives can be used with normal glass (*n* = 1.53) coverslip and oil, they are the most useful for total internal reflection, except for cells which have particularly dense organelles (e.g. adipocytes which contain a lot of lipid droplets), and they are available with three range of magnification: 60×, 100× and 150×. To overcome the resolution limit imposed by the charge-coupled device camera’s (CCD) pixel size it is advisable to use the highest objective magnification to enhance small structures, as is the case for insulin dense-core secretory vesicle. These objectives also use a correction collar to compensate for coverslip thickness and temperature.

##### 2.2.1.2 Laser source

A laser with a total visible output in the 30 mW (or greater) range should be adequate for most TIRF imaging applications using fluorescent proteins such as GFP. In our case, we use a 50 mW air-cooled single line 488 nm argon-ion laser (Spectra Physics) or 30 mW diode pumped solid-state (DPSS) 488 nm laser (Yokokawa). Laser light is usually delivered using optical fibres, but mirror based systems can provide a less expensive alternative [30]. Laser illumination produces interference fringes which are manifested as intensity variations over the sample area. For critical applications, it may be advisable to rapidly “jiggle” the beam (i.e. commercially available optical fiber mode scrambler) or to compute a normalization of sample digital images against a control digital image of a uniform concentration of fluorophores.

##### 2.2.1.3 Camera

There are many types of charge-coupled device (CCD) camera now available for detecting TIRF images. A cooled-CCD, electron bombardment (EB)-CCD, or electron multiplyer (EM)-CCD, or CCD camera boosted with generation IV (Gen-IV) image intensifier should be enough for most of TIRF imaging. EB-CCD camera and CCD camera boosted with Gen-IV image intensifier (i.e. VS4-1845, VideoScope) is suitable for video rate (33 ms per frame) TIRF imaging. Cooled-CCD and EM-CCD camera enable us to detect much faster rate of TIRF image (∼1 ms per frame, but it is up to the camera’s property). In our case, we used cooled-CCD (Imago, Till Photonics), EM-CCD camera (C9100-02, Hamamatsu Photonics) or a Roper Scientific 512 cascade camera to detect insulin exocytosis from pancreatic β-cells.

##### 2.2.1.4 “Off the Peg” systems

Complete, multiline TIRF systems are available from several manufacturers including Olympus (http://www.olympusmicro.com/), Nikon (http://www.microscopyu.com/), Leica (http://www.leica-microsystems.com), Zeiss (http://www.zeiss.com) and TILL Photonics (Agilent Technologies, http://www.zeiss.com).

#### 2.2.2 Step-by-step protocol

A step-by-step guide for setting up TIRF in a standard fluorescence microscope has been published elsewhere [31]. Details are also available in manuals from companies to set-up your system. So, in this section, we will just briefly describe the protocol avoiding “technical jargon” as much as possible.

**Fig. 3:**
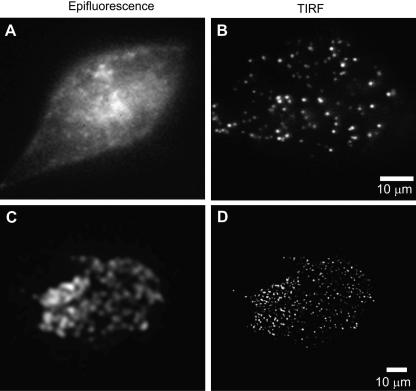
Epifluorescence versus objective lens-type total internal reflection fluorescence. Images were captured with Olympus 1.45 NA 100× objective lens and an argon ion laser source of wavelength 488 nm, using the side-port configuration depicted in Fig. 2. (A and B) PC12 cells containing secretory vesicle marker neuropeptide Y-Venus (NPY-Venus). (C and D) PC12 cells containing secretory vesicle marker rabphilin-mRFP. The images were recorded by a cooled monochrome CCD camera (Imago, Till Photonics) in A and B, and recorded by EM-CCD camera (C9100-02, Hamamatsu Photonics) in C and D.

1. Place a drop of immersion oil on the objective, put a bare coverslip on the microscope stage, and focus on the coverslip (upper surface).
2. Try to remove everything between the coverslip and the room ceiling (i.e. tilt backwards the epi-fluorescence upper stage of the microscope). Collimate the beam laser (every time the objective is changed). The laser should be introduced along the optical axis, and an area of laser illumination will be seen on the room ceiling (straight up). Never look into the objective or into the laser beam during this procedure because exposure of eyes to laser beam may be dangerous. It is therefore advisable to use safety goggles.
3. While observing the laser beam on the room ceiling, adjust the size (focus) of the laser spot: try to minimize it.
4. The next step will be to achieve and fix the right angle to get TIRF. Basically, depending on the systems, you will need to turn a knob to move the focal point of the laser beam to left or right (or to forward or backward). As an example, if we decide to move the laser beam to the left, the angle will move, and the laser beam on the ceiling will move to the left wall of the room, until it disappears. When it starts to disappear you are close to the total internal reflection. The steps above were done by watching the laser beam on the wall, rather than through the eyepiece of the microscope.
5. To fine-tune the total internal reflection either use fluorescent beads or your cell sample.
• *Fluorescent beads.* Place a droplet of water containing fluorescent beads (∼0.5 μm diameter) directly on the coverslip. One waits a few minutes for the beads to reach the upper surface of the coverslip, and then go to step 6. However, because they are not heavy it will take time for the beads to reach the upper side of the coverslip. Moreover, because of the Brownian motion it will be difficult to focus the upper side of the coverslip while watching beads.
• *Cell sample*. Because of the problems depicted above, we recommend to replace the bare coverslip with your cell sample coverslip (cells containing fluorescent vesicles adherent to a coverslip).
6. Next watch the fluorescence through the eyepiece of the microscope, you should still see a similar pattern as in epifluorescence mode (Fig. 3A and C). Then, by turning again gently the laser position adjustment knob one can fine-tune the total internal reflection (Fig. 3B and D). Once total internal reflection is achieved, further increases in off-axis position serve to increase the incidence angle at the sample and thereby decrease the depth of the evanescent field.

### 2.3 Fluorescent probes for detecting secretion

Here, we will describe some probes that can be used to detect exocytosis. This list is not exhaustive as it depends as well on the cell type you want to study (Section 2.3.2).

#### 2.3.1 Chemical probes

##### 2.3.1.1 Acridine orange

TIRF observation of insulin vesicle motion in β cells was first reported by us [16]. We used a weak base organic fluorescent dye acridine orange, which accumulates in acidic compartments in cells [16,27,32]. This was achieved since mature hormone-containing vesicles maintain an acidic lumen (pH 5.0–6.0) [33], resulting from the activity of a vacuolar H^+^-ATPase [34]. However, acridine orange also labels other acidic intracellular compartments (i.e. lysosomes, early and late endosomes, autophagosomes).

##### 2.3.1.2 FM dyes

The family of fluorescent styryl compounds known as FM dyes has been useful for the study of exocytosis, endocytosis, and vesicle trafficking. They are used to selectively visualise plasma membrane as they become fluorescent when they insert into external leaflet of the surface membrane. Further details on these probes are found in the following review [35].

#### 2.3.2 Gene transfer

Because genetically-encoded probes (fluorescent proteins) can be introduced by gene-transfer techniques, they can extract exocytotic response signals from an intact secreting cell more efficiently than conventional chemical fluorescent dyes. For example, whereas conventional optical imaging of endocrine cells stained with acidic compartment-sensitive chemical dyes is a non-invasive technique for visualising the exocytosis, this technique collects signals from exocytosis of other acidic organelles, as mentioned above. In contrast, the selective introduction of genetically-encoded probes into specific subcellular compartments (i.e. hormone-containing vesicles) has enabled the elimination of other acidic organelle’s signals.

##### 2.3.2.1 pH-insensitive fluorescent proteins

One can use pH-insensitive (or less sensitive) fluorescent protein variants which are fused to the peptide hormone. This requires efficient maturation and folding property of fluorescent proteins, since the inside of secretory vesicles is acidic. Poor folding of a fused fluorescent protein variants result in a non-fluorescent chimera. Moreover, the accumulation of a poor folded protein into the granule will induce a decrease of the global fluorescence and may disturb the exocytosis and cellular homeostasis [36]. For example, targeting of normal green fluorescent protein (GFP) or wild type DsRed-fused chromogranin A or insulin chimeras to secretory vesicles can show mistargeting [9,37–39].

Maturation of fluorescent proteins is a multistep process that consists of cyclization, dehydration, and oxidation [40]. Recently, several bright fluorescent proteins that mature efficiently have been developed. These include Citrine [41] and Venus [42], two yellow-emitting variants of *Aequorea victoria* GFP, and several red-emitting variants of DsRed [43,44]. These fluorescent protein variants also facilitate protein folding when incorporated into probes [42]. Therefore, we have used Venus or monomeric red fluorescent protein (mRFP) fused with neuropeptide Y as a secretory vesicle marker [17,45]. The targeting efficiency of NPY-Venus was compared with endogenous CgA by immunocytochemical analysis of fixed cells. Examined in adrenal chromaffin tumor derivative PC12 cells as a neuroendocrine model cells, over 90% of NPY-Venus-expressing vesicles were well colocalized with CgA. We have also targeted to granules GFP fused to phogrin (phosphatase on the granule of insulinoma cells [46]) [39], thus GFP is not expressed inside the granule, but at the granular membrane facing the cytosol. Others have fused insulin-C-peptide to emerald green, EGFP, or bright mutant of DsRed [18] or the islet amyloid polypeptide to EGFP [18,47].

##### 2.3.2.2 pH-sensitive fluorescent protein variants

An alternative method for the visualisation of secretion is by using pH-sensitive fluorescent protein variants which target to secretory vesicle membrane proteins. Miesenbock et al. [48] originally developed pH-sensitive GFP (pHluorin), which is brightly fluorescent at approximately pH 7.4, but not at pH 5.0. Therefore, pHluorin targeted to the secretory vesicle lumen is able to distinguish between secretory and non-secretory vesicles. This is feasible since, after secretion, the pH of the secretory vesicle lumen becomes alkaline in the extracellular environment (∼pH 7.4). The first example of this approach employed synapto-pHluorin [49]. This protein reports the release of neurotransmitter, i.e., the exocytosis of synaptic vesicles. A pHluorin is fused to the synaptic vesicle luminal protein of synaptobrevin2 (VAMP2). As the lumenal pH increases from acidic to neutral, the signal of synapto-pHluorin increases by about 20-fold. A pHluorin has also been fused to insulin or phogrin to detect exocytosis from pancreatic β-cell lines [50,51]. In the case of pH-sensitive fluorescent protein variants, the release event will be accompanied by a flash of the released probe (increase in fluorescence due to the alkaline extracellular environment), and thus can distinguish unambiguously between a release event and a vesicle that goes to the plasma membrane and go back without secreting its content.

All of the above genetically-encoded probes must be introduced by gene-transfer techniques in cells, which can be achieved by transfection using conventional approaches (Ca^2+^–phosphate, lipofectamine, micro-injection, electroporation, gene gun, etc.). However, none of these techniques have been successful in our hands for protein expression in primary pancreatic β-cells, thus the generation of an adenovirus or other viral vector is highly recommended [9,52].

### 2.4 Data analysis

**Fig. 4:**
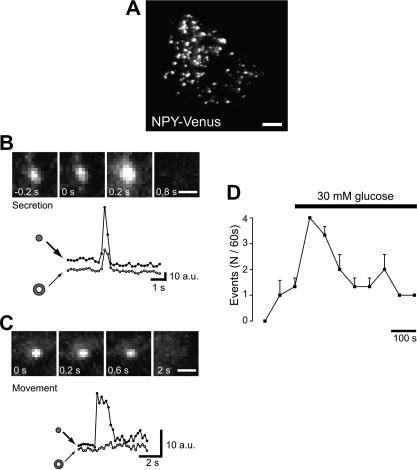
Analysis of secretion by TIRF microscopy. (A) Evanescent wave image of NPY-Venus fluorescence in a live MIN6 pancreatic β cell. The scale bar represents 5 μm. (B, top) Sequential images of a single vesicle observed after high [K^+^] stimulation. The third image shows a diffuse cloud of the NPY-Venus fluorescence, and the final image shows an abrupt disappearance of the fluorescent spot. (B and C, bottom) Time course of the fluorescence changes measured in the small circles enclosing fluorescent spots (filled symbols) and for concentric annuli around the circles (open symbols) of two different vesicles. The ordinate is given in arbitrary units of brightness. (C, top) Sequential images of a single vesicle observed after stimulation with 50 mM KCl without showing any diffuse cloud of the NPY-Venus fluorescence. The third image does not show any cloud of the dye, whereas the final image shows an abrupt disappearance. These events typically exhibited a much slower time course (approximately 3 s to reach peak fluorescence) than those showing in B and reflects the approach of vesicles to the plasma membrane without exocytosis (i.e. vesicle movement or retrieval). The scale bars represents 1 μm. (D) Effect of glucose on exocytosis as reported with NPY-Venus in pancreatic β cells. The NPY-Venus spots shown in B was counted manually as secretion events every 60 s and plotted against time. Stimulation with high glucose concentrations (30 mM) caused a marked increase in the number of secretion.

To analyze TIRF imaging data, single exocytotic events are selected manually, and the average fluorescence intensity of individual vesicle in a 0.7 × 0.7 μm^2^ placed over the vesicle center calculated using MetaMorph software (version 6.3, Universal Imaging Corporation, Downingtown, PA). To distinguish between fusion events and vesicle movement (i.e. vesicles pause at the plasma membrane and then move back inside the cell without fusing), we focus on fluorescence changes just before the disappearance of fluorescent signals. When there is a fusion event, a rapid transient increase in fluorescence intensity (to a peak intensity 1.5 times greater than the original fluorescence intensity within 1 s) is usually observed, whereas when vesicles move, the fluorescence intensity gradually decreases to the background level (Fig. 4). The number of fusion events during a 5-min period can be counted manually, based on the above criteria [9,17,53].

## 3 Concluding remarks: alternative systems

In addition to TIRF, at least two further systems can be used to image secretory granule movement and fusion: confocal and two photon microscopy.

### 3.1 Confocal microscopy

In a conventional fluorescence microscope, the fluorescence signal is detected from the entire cell, thus all planes are visible, not just the focused plane. The confocal microscopy excludes the out-of-focus light (above and below the plane of focus) by using confocal pinholes. The final image will thus display only the focused object (i.e. one slice of the cell).

The main advantage of confocal microscopy is that release events can be monitored over the entire cell surface (time lapse of Z series) most convenient using Nipkow spinning disc optics [54] which allows more rapid acquisition of images (i.e. a complete stack of 10–15 images in <1 s) and less bleaching than laser scanning devices [39]. It is thus possible to reconstitute exocytotic events overall the entire cell surface in three dimensions *post facto*, as the recording is not restricted to the bottom of the cell only as in TIRF. Nevertheless, for clonal β-cells we showed using the spinning disc approach above that events occur with roughly equal probability over the entire cell surface [54] and the combination of membrane capacitance measurements and TIRF in chromaffin cells showed that secretion at the footprint of the cells is correlated with the overall secretion of the cell [55]. Thus, secretion is likely to be similar at the bottom or at the top of the cell, at least in non-polarized cells.

However, confocal microscopy also has disadvantages in comparison to TIRF. The optical sectioning is usually thicker (∼500 nm), corresponding to at least two layers of granules in β-cells, versus one layer in TIRF. Furthermore, in other applications such as fluorescence recovery after photobleaching (FRAP), or photoconversion, the illumination of a thin section is clearly preferable. Last but not least, TIRF can be adapted to standard microscopes, and it is much less expensive than a confocal microscope. Nevertheless, the choice of the system (i.e. confocal versus TIRF) is highly dependent on the question to be answered.

### 3.2 Two-photon microscopy

Two-photon microscopy has many advantages, and has been used to detect exocytosis [56,57]. The two-photon absorption takes place at the focus of the laser beam, allowing a three dimensional resolution, and can be used to image inside organs. However, the optical sectioning is still thicker than TIRF, and multiphoton systems are considerably more expensive.
